# Association of KCTD10, MVK, and MMAB polymorphisms with dyslipidemia and coronary heart disease in Han Chinese population

**DOI:** 10.1186/s12944-016-0348-7

**Published:** 2016-10-04

**Authors:** Jie Sun, Yun Qian, Yue Jiang, Jiaping Chen, Juncheng Dai, Guangfu Jin, Jianming Wang, Zhibin Hu, Sijun Liu, Chong Shen, Hongbing Shen

**Affiliations:** 1 Department of Epidemiology, School of Public Health, Nanjing Medical University, 101 Longmian AV., Nanjing, Jiangsu 211166 China; 2 Department of Chronic Non-communicable Disease Control, Wuxi Center for Disease Control and Prevention, Wuxi, 214023 China; 3 Department of Social Medicine and Health Education, School of Public Health, Nanjing Medical University, 101 Longmian AV., Nanjing, Jiangsu 211166 China

**Keywords:** *MVK*, *MMAB*, *KCTD10*, HDL-C, Coronary heart disease

## Abstract

**Background:**

Several genome-wide association studies have discovered novel loci at chromosome 12q24, which includes mevalonate kinase (*MVK*), methylmalonic aciduria (cobalamin deficiency) cbIB type (*MMAB*), and potassium channel tetramerization domain-containing 10 (*KCTD10*), all of which influence HDL-cholesterol concentrations. However, there are few reports on the associations between these polymorphisms and HDL-C concentrations in Chinese population. This study aimed to evaluate the associations between functional polymorphisms in three genes (*MVK*, *MMAB* and *KCTD10*) and HDL-C concentrations, as well as coronary heart disease (CHD) susceptibility in Chinese individuals.

**Methods:**

We systematically selected and genotyped 18 potentially functional polymorphisms in *MVK*, *MMAB* and *KCTD10* by using the TaqMan OpenArray Genotyping System in a Chinese population including 399 dyslipidemia cases, 697 CHD cases and 465 controls. Multivariate logistic regression analyses were performed to estimate the relationship between the genotypes and dyslipidemia, CHD risk with adjustment of relevant confounders.

**Results:**

Among six polymorphisms showing significant associations with dyslipidemia, the minor alleles of rs11066782 in *KCTD10*, rs11613718 in *KCTD10* and rs11067233 in *MMAB* were significantly associated with a decreased risk of CHD (additive model: OR = 0.71, 95 % CI = 0.53–0.97, *P* = 0.029 for rs11066782; OR = 0.73, 95 % CI = 0.54–0.99, *P* = 0.044 for rs11613718 and OR = 0.57, 95 % CI = 0.40–0.80, *P* = 0.001 for rs11067233). Further combined analysis showed that individuals carrying “3-4” favorable alleles presented a 62 % (OR = 0.38, 95 % CI = 0.21–0.66) decreased risk of CHD compared with those carrying “0–2” favorable alleles.

**Conclusions:**

These findings suggest that rs11066782 in *KCTD10*, rs11613718 in *KCTD10* and rs11067233 in *MMAB* may contribute to the susceptibility of CHD by altering plasma HDL-C levels in Han Chinese.

**Electronic supplementary material:**

The online version of this article (doi:10.1186/s12944-016-0348-7) contains supplementary material, which is available to authorized users.

## Background

Coronary heart disease (CHD) is one of the leading causes of morbidity and mortality in the world [1]. In China, it is reported that more than 700,000 people die from CHD each year [2]. CHD is a complex and multifactorial disorder caused by various environmental and genetic factors [3, 4]. Clinical and epidemiological studies have demonstrated that the presence of low levels of high-density lipoprotein-cholesterol (HDL-C) in plasma increases the risk of developing CHD [5–8]. The mechanism by which HDL-C confers protection against atherosclerosis includes reverse cholesterol transport from peripheral tissues to the liver [9], inhibition of low-density lipoprotein-cholesterol (LDL-C) oxidation, and stabilization of the production of prostacyclin [10]. While smoking, diet and physical activity have a role in determining plasma HDL-C concentrations, family and twin studies have shown that about half of the variation in this trait is genetically determined [11, 12]. In 2008, a genome-wide association study (GWAS) conducted in populations of European descent discovered novel loci at chromosome 12q24, which includes mevalonate kinase (*MVK*), methylmalonic aciduria (cobalamin deficiency) cbIB type (*MMAB*), and potassium channel tetramerization domain-containing 10 (*KCTD10*), all of which influence HDL-C concentrations [13]. This association has been consistently replicated in the subsequent studies [14–16]. *MVK* encodes the mevalonate kinase, which catalyzes an early step in the biosynthesis of cholesterol [17]. *MMAB* encodes the enzyme which catalyzes the formation of adenosylcobalamin, a critical factor for degradation of cholesterol [17]. *KCTD10*, which is close to *MVK* and *MMAB* genes, has shown to contribute to the susceptibility of obesity, diabetes and atherosclerosis [18]. Therefore, *MVK*, *MMAB* and *KCTD10* genes may be candidates as susceptibility genes modulating HDL-C concentrations and then affect the risk of dyslipidemia and CHD.

To determine whether polymorphisms in *MVK*, *MMAB* and *KCTD10* are independently associated with the risk of dyslipidemia and CHD, we conducted a case–control study with 399 dyslipidemia cases and 465 controls in Han Chinese. In addition, for those loci showing significant associations with dyslipidemia, we further evaluated the associations between these polymorphisms and CHD risk including 697 CHD cases and 465 controls.

## Methods

### Study population

In this study, we performed a two-stage case–control study. The first-stage analysis was designed to discover the suggestive variants associated with dyslipidemia in a Chinese population consisting of 399 cases and 465 controls from a community based cohort study of non-infectious diseases in Changzhou and Nantong cities, Jiangsu Province, China. In this study, eligible subjects aged over 30 years old were enrolled in 2004 and 2007, and the baseline information including demographic, disease history and risk factors for chronic disease was obtained and a detailed clinical examination was conducted. Physical examinations, including measurements of height, weight and blood pressure as well as laboratory tests to measure total cholesterol (TC), triglycerides (TG), HDL-C and fasting plasma glucose concentrations, were performed for each subject. Fasting blood samples for routine laboratory examinations were obtained early in the morning after an overnight fast. All biochemical parameters were measured enzymatically on an auto-analyzer (Hitachi 7180 Biochemistry Auto-analyzer, Japan) according to the manufacturer’s instructions.

According to the Guidelines on Prevention and Treatment of dyslipidemia in Chinese Adults [19], 399 subjects with HDL-C <1.04 mmol/L were defined as cases with dyslipidemia (Low-HDL cholesterolemia), while 465 subjects not meeting this criteria were randomly selected and matched with the cases for age and sex. Subjects were excluded from the study if they had a history of diabetes, coronary heart disease or cancer, or those who were taking lipid-lowering medications.

The second-stage analysis was to determine whether the loci that influence the HDL-C levels in the first-stage also have an effect on CHD susceptibility, which consisted of 697 CHD cases from People's Hospital of Yixing City, Jiangsu Province, China and 465 controls defined in the first-stage. In brief, CHD cases were defined as having angiographic coronary stenosis with ≥50 % lumen reduction in at least one major epicardial coronary artery. All patients were genetically unrelated ethnic Han Chinese from Yixing city. After informed consent was obtained, the information of demographic characteristics, risk factors for CHD, history of vascular events and clinical diagnosis were written from the clinical records. A 5-ml venous blood sample was collected from each patient.

### SNP selection

Based on the public HapMap SNP database (phase II + III Feb 09, on NCBI B36 assembly, dbSNP b126) and the HaploView 4.2 software, common SNPs (MAF ≥0.05) in the three genes (*MVK*, *MMAB* and *KCTD10*) were screened in gene regions (including 2-kb up-stream region of each gene) in Chinese Han population. A total of 21 potentially functional SNPs were selected after the prediction by using SNPinfo Web Server (http://snpinfo.niehs.nih.gov/) which is a comprehensive web-based tool designed to select SNPs based on GWAS results, linkage disequilibrium, and predict potential functional characteristics of polymorphisms. Besides, in order to validate the findings of GWAS [13, 15], rs2338104 and rs7134594 were also included. However, three SNPs were excluded for the failed probe design and two SNPs were removed due to genotyping failure (call rates <95 %). As a result, 18 loci were finally included in the current study. The linkage disequilibrium (LD) association for the 18 loci was further analyzed (Additional file 1: Figure S1).

### Genotyping

Genomic DNA was isolated from leucocytes of venous blood by proteinase K digestion and phenol/chloroform extraction. All SNPs were genotyped by using the TaqMan OpenArray Genotyping System (Applied Biosystems lnc, USA). Fluorescence-based polymerase chain reaction (PCR) reagents were used to provide qualitative detection of targets using post-PCR (endpoint) analysis. A total volume of 5 μl with 2.5 μl TaqMan OpenArray Master Mix and 2.5 μl normalized human DNA sample (50 ng/μl) were loaded and amplified on customized arrays following the manufacturer’s instructions. Each 48-sample array chip contained two NTCs (no template controls). All 18 SNPs were successfully genotyped with call rates >95 %. Additionally, 10 % of samples were randomly selected for genotyping in duplicates and the results demonstrated a high degree of concordance (>99 %) among the duplicate pairs.

### Statistical analysis

Data were shown as mean ± standard deviation (SD) or n (%). Differences in the distributions of demographic characteristics, selected variables and genotypes frequencies between cases and controls were analyzed by *χ* ^2^ test or Student *t* test. Hardy-Weinberg equilibrium was tested by a goodness-of-fit *χ* ^2^ test to compare the observed genotype frequencies to expected frequencies among the control subjects. Associations between the genotypes and dyslipidemia, CHD risk were estimated by computing the odds ratios (OR) and 95 % confidence intervals (CIs) from logistic regression analyses with adjustment for age, sex, smoking status and body mass index (BMI). The Chi-square-based Q test was used to test the heterogeneity of associations between subgroups. The effects of selected loci on plasma TC, TG and HDL-C concentrations were evaluated using the multiple linear regression method with adjustment for age, sex, smoking status and BMI. The significance level was set at *P* < 0.05 and *P* values were given for two-sided tests. All statistical analyses were performed using R software (Version 3.0.2, 2013-09-25; R Foundation for Statistical Computing, http://www.cran.r-project.org/). Data in this study was available (Additional file 2).

## Results

### Characteristics of the subjects

The characteristics of the subjects are shown in Table 1. There were no statistically significant differences between the cases with dyslipidemia and the controls in terms of age, sex, and smoking status. BMI (*P* < 0.0001), TC (*P* < 0.0001) and TG (*P* < 0.0001) were significantly higher in cases with dyslipidemia compared with the controls. Mean HDL-C concentration in cases with dyslipidemia (0.81 ± 0.18 mmol/L) was significantly lower than that in controls (1.81 ± 0.31 mmol/L). Additionally, the mean age of the CHD cases (67.61 ± 11.13 years) were significantly higher than that of controls (49.48 ± 12.46 years). The proportion of male in the CHD cases (57.96 %) was higher as compared with those in controls (46.45 %) (*P* = 0.0001), whereas similar distributions of smokers were observed between groups (*P* = 0.051). Besides, TC (*P* < 0.0001) and TG (*P* < 0.0001) were significantly higher in the CHD cases than that in controls. HDL-C (*P* < 0.0001) concentration was significantly lower in the CHD cases compared with the controls.

**Table 1:** Characteristics of the subjects

Variables	Controls	Cases with dyslipidemia	Cases with coronary heart disease
	(*N* = 465)	(*N* = 399)	(*N* = 697)
Age (years)	49.48 ± 12.46	48.97 ± 12.68	67.61 ± 11.13
BMI (kg/m^2^)	21.98 ± 3.01	25.34 ± 3.23	
Sex			
Male	216 (46.45)	193 (48.37)	404 (57.96)
Female	249 (53.55)	206 (51.63)	293 (42.04)
Smoking			
Ever	148 (31.83)	131 (33.68)	185 (26.54)
Never	317 (68.17)	258 (66.32)	512 (73.46)
TC (mmol/L)	3.94 ± 0.60	4.37 ± 0.92	4.48 ± 2.05
TG (mmol/L)	0.75 ± 0.22	3.16 ± 1.33	1.61 ± 1.48
HDL-C (mmol/L)	1.81 ± 0.31	0.81 ± 0.18	1.18 ± 0.31

Data were shown as mean ± standard deviation (SD) or *n* (%)

*BMI* body mass index, *TC* total cholesterol, *TG* triglycerides, *HDL-C* high-density lipoprotein-cholesterol

### Association analyses for dyslipidemia

The association results of 17 SNPs in codominant and additive models were described in Table 2. The SNP rs4499061 deviated from Hardy-Weinberg equilibrium among controls (*P* < 0.05) and was excluded from subsequent analyses. After adjustment of age, sex, smoking status and BMI, five polymorphisms (*KCTD10* rs1477117, *KCTD10* rs11066782, *KCTD10* rs11613718, *KCTD10* rs11615336 and *MMAB* rs12817689) showed significant associations with dyslipidemia risk in the codominant model (minor homozygote vs. major homozygote) and *MMAB* rs11067233 was significantly associated with dyslipidemia risk in the additive model.

**Table 2:** Summary results of associations between 17 potentially functional SNPs in three genes (*KCTD10*, *MVK* and *MMAB*) and risk of dyslipidemia

Gene	SNP	Allele^a^	Cases^b^	Controls^b^	MAF	HWE^c^	Codominant model^d^		Additive model^d^
			(*N* = 399)	(*N* = 465)	(Cases/controls)		*P*_het_	*P*_hom_	*P*_add_
*KCTD10*	rs1477117	G/A	279/115/1	326/123/10	0.148/0.156	0.686	0.349	0.025	0.791
	rs11066782	C/T	283/113/1	311/135/11	0.145/0.172	0.414	0.929	0.014	0.174
	rs11613718	C/T	272/120/1	308/139/9	0.155/0.172	0.138	0.762	0.038	0.521
	rs11615336	C/A	282/101/1	317/120/10	0.134/0.157	0.730	0.931	0.024	0.288
	rs7295954	C/T	322/60/1	381/69/0	0.081/0.077	0.078	0.399	–	0.323
	rs1045582	G/T	284/104/2	321/124/6	0.138/0.151	0.118	0.888	0.112	0.459
*MVK*	rs3759387	G/T	297/97/3	330/126/7	0.130/0.151	0.194	0.990	0.303	0.687
	rs2287218	C/T	279/112/4	324/122/10	0.152/0.156	0.707	0.494	0.095	0.878
*MMAB*	rs12817689	A/G	284/105/3	319/113/10	0.142/0.150	0.998	0.614	0.047	0.635
	rs2241201	C/G	238/138/11	288/148/21	0.207/0.208	0.722	0.358	0.268	0.928
	rs877710	C/G	194/177/26	219/201/40	0.288/0.305	0.522	0.656	0.330	0.714
	rs11067233	C/G	315/74/2	329/118/8	0.100/0.147	0.486	0.072	0.145	0.026
	rs9593	A/T	187/162/26	212/196/39	0.285/0.306	0.506	0.958	0.333	0.539
	rs11067227	C/T	316/67/2	333/104/7	0.092/0.133	0.729	0.116	0.295	0.065
	rs7134594	C/T	187/178/26	213/196/40	0.294/0.307	0.286	0.786	0.364	0.665
	rs11831226	A/C	320/58/2	391/71/0	0.082/0.077	0.074	0.491	–	0.342
	rs8228	A/G	322/63/0	380/69/0	0.082/0.077	0.078	0.349	–	0.349

*MAF* minor allele frequency

^a^Major/minor allele

^b^Major homozygote/heterozygote/rare homozygote

^c^Hardy-Weinberg equilibrium test among controls

^d^Logistic regression with adjustment for sex, age, smoking and BMI were used to test associations in codominant (*P*_het_: heterozygote vs. major homozygote; *P*_hom_: minor homozygote vs. major homozygote) and additive (*P*_add_: minor homozygote vs. heterozygote vs. major homozygote) models

### Association analyses for plasma lipid concentrations

We examined the effect of these six loci on plasma TC, TG, or HDL-C concentrations, respectively, using a linear regression model with adjustment for age, sex, smoking status and BMI (Table 3). We found significant associations between rs11066782, rs11613718, rs11615336 and rs12817689 and TC (codominant model: *P* = 0.035, 0.025, 0.005 and 0.038, respectively), between rs1477117, rs11066782 and rs11615336 and TG (recessive model: *P* = 0.028, 0.015 and 0.031, respectively), between rs1477117, rs11066782, rs11613718 and rs11615336 and HDL-C (recessive model: *P* = 0.024, 0.007, 0.031 and 0.025, respectively), between rs11067233 and HDL-C (dominant model: *P* = 0.047). Additionally, the minor alleles of these associated SNPs were consistently associated with lower TC or TG levels and higher HDL-C levels.

**Table 3:** Associations between selected single nucleotide polymorphisms and plasma lipid concentrations

Polymorphisms	TC (mmol/L)	*P* ^a^	TG (mmol/L)	*P* ^a^	HDL-C (mmol/L)	*P* ^a^
rs1477117 (G > A)						
GG (*n* = 605)	4.17 ± 0.80	0.143	1.86 ± 1.49	0.848	1.34 ± 0.55	0.456
GA (*n* = 238)	4.11 ± 0.80		1.91 ± 1.58		1.35 ± 0.59	
AA (*n* = 11)	3.68 ± 0.63		0.94 ± 0.50		1.68 ± 0.50	
GG (*n* = 605)	4.17 ± 0.80	0.255	1.86 ± 1.49	0.733	1.34 ± 0.55	0.812
GA + AA (*n* = 249)	4.09 ± 0.80		1.87 ± 1.56		1.36 ± 0.59	
GG + GA (*n* = 843)	4.15 ± 0.80	0.081	1.88 ± 1.52	0.028	1.34 ± 0.56	0.024
AA (*n* = 11)	3.68 ± 0.63		0.94 ± 0.50		1.68 ± 0.50	
rs11066782 (C > T)						
CC (*n* = 594)	4.19 ± 0.80	0.035	1.90 ± 1.50	0.319	1.33 ± 0.55	0.065
CT (*n* = 248)	4.07 ± 0.79		1.85 ± 1.58		1.38 ± 0.60	
TT (*n* = 12)	3.82 ± 0.77		0.93 ± 0.48		1.71 ± 0.49	
CC (*n* = 594)	4.19 ± 0.80	0.052	1.90 ± 1.50	0.653	1.33 ± 0.55	0.197
CT + TT (*n* = 260)	4.06 ± 0.79		1.80 ± 1.56		1.39 ± 0.59	
CC + CT (*n* = 842)	4.15 ± 0.80	0.202	1.89 ± 1.52	0.015	1.34 ± 0.56	0.007
TT (*n* = 12)	3.82 ± 0.77		0.93 ± 0.48		1.71 ± 0.49	
rs11613718 (C>T)						
CC (*n* = 580)	4.18 ± 0.79	0.025	1.88 ± 1.50	0.599	1.33 ± 0.55	0.243
CT (*n* = 259)	4.07 ± 0.78		1.86 ± 1.56		1.37 ± 0.59	
TT (*n* = 10)	3.71 ± 0.66		0.96 ± 0.52		1.70 ± 0.53	
CC (*n* = 580)	4.18 ± 0.79	0.042	1.88 ± 1.50	0.914	1.33 ± 0.55	0.461
CT + TT (*n* = 269)	4.05 ± 0.78		1.83 ± 1.55		1.38 ± 0.59	
CC + CT (*n* = 839)	4.15 ± 0.79	0.131	1.88 ± 1.52	0.053	1.34 ± 0.56	0.031
TT (*n* = 10)	3.71 ± 0.66		0.96 ± 0.52		1.70 ± 0.53	
rs11615336 (C > A)						
CC (*n* = 599)	4.19 ± 0.80	0.005	1.90 ± 1.51	0.191	1.34 ± 0.55	0.275
CA (*n* = 221)	4.03 ± 0.75		1.80 ± 1.53		1.36 ± 0.60	
AA (*n* = 11)	3.68 ± 0.63		0.94 ± 0.50		1.68 ± 0.50	
CC (*n* = 599)	4.19 ± 0.80	0.009	1.90 ± 1.51	0.392	1.34 ± 0.55	0.546
CA + AA (*n* = 232)	4.02 ± 0.75		1.76 ± 1.51		1.38 ± 0.60	
CC + CA (*n* = 820)	4.15 ± 0.79	0.082	1.87 ± 1.51	0.031	1.34 ± 0.56	0.025
AA (*n* = 11)	3.68 ± 0.63		0.94 ± 0.50		1.68 ± 0.50	
rs12817689 (A > G)						
AA (*n* = 603)	4.18 ± 0.80	0.038	1.89 ± 1.50	0.733	1.34 ± 0.55	0.423
AG (*n* = 218)	4.06 ± 0.78		1.90 ± 1.58		1.35 ± 0.60	
GG (*n* = 13)	3.88 ± 0.67		1.34 ± 1.20		1.54 ± 0.57	
AA (*n* = 603)	4.18 ± 0.80	0.051	1.89 ± 1.50	0.995	1.34 ± 0.55	0.669
AG + GG (*n* = 231)	4.05 ± 0.77		1.87 ± 1.56		1.36 ± 0.60	
AA + AG (*n* = 821)	4.14 ± 0.79	0.278	1.89 ± 1.52	0.172	1.34 ± 0.56	0.099
GG (*n* = 13)	3.88 ± 0.67		1.34 ± 1.20		1.54 ± 0.57	
rs11067233 (C > G)						
CC (*n* = 644)	4.15 ± 0.80	0.392	1.94 ± 1.54	0.123	1.32 ± 0.56	0.049
CG (*n* = 192)	4.17 ± 0.82		1.68 ± 1.46		1.44 ± 0.55	
GG (*n* = 10)	4.10 ± 0.63		1.38 ± 1.12		1.51 ± 0.42	
CC (*n* = 644)	4.15 ± 0.80	0.347	1.94 ± 1.54	0.137	1.32 ± 0.56	0.047
CG + GG (*n* = 202)	4.17 ± 0.81		1.66 ± 1.44		1.44 ± 0.55	
CC + CG (*n* = 836)	4.15 ± 0.80	0.952	1.88 ± 1.52	0.482	1.35 ± 0.56	0.591
GG (*n* = 10)	4.10 ± 0.63		1.38 ± 1.12		1.51 ± 0.42	

*TC* total cholesterol, *TG* triglycerides, *HDL-C* high-density lipoprotein-cholesterol

^a^Multiple linear regression with adjustment for sex, age, smoking and BMI

### Association analyses for CHD

To further evaluate the associations of the significant polymorphisms and CHD risk, we genotyped these six promising SNPs in another 697 CHD cases. As shown in Table 4, after adjustment of age, sex and smoking status, the minor alleles of rs11066782 in *KCTD10*, rs11613718 in *KCTD10* and rs11067233 in *MMAB* were significantly associated with a decreased risk of CHD (additive model: OR = 0.71, 95 % CI = 0.53–0.97, *P* = 0.029 for rs11066782; OR = 0.73, 95 % CI = 0.54–0.99, *P* = 0.044 for rs11613718 and OR = 0.57, 95 % CI = 0.40–0.80, *P* = 0.001 for rs11067233).

**Table 4:** Associations between selected single nucleotide polymorphisms and risk of coronary heart disease

Gene	SNP	Cases^a^	Controls^a^	MAF	Heterozygote vs. major homozygote		Minor homozygote vs. major homozygote		Additive model	
		(*N* = 697)	(*N* = 465)	(Cases/controls)	OR (95 % CI)^b^	*P* ^b^	OR (95 % CI)^b^	*P* ^b^	OR (95 % CI)^b^	*P* ^b^
*KCTD10*	rs1477117	521/156/16	326/123/10	0.136/0.156	0.70 (0.49–1.00)	0.048	1.13 (0.39–3.33)	0.820	0.79 (0.58–1.08)	0.135
	rs11066782	517/160/15	311/135/11	0.137/0.172	0.62 (0.43–0.88)	0.007	1.02 (0.35–2.94)	0.975	0.71 (0.53–0.97)	0.029
	rs11613718	494/160/15	308/139/9	0.142/0.172	0.63 (0.44–0.90)	0.010	1.12 (0.37–3.35)	0.840	0.73 (0.54–0.99)	0.044
	rs11615336	502/166/16	317/120/10	0.145/0.157	0.74 (0.52–1.06)	0.104	1.12 (0.38–3.30)	0.835	0.83 (0.61–1.12)	0.223
*MMAB*	rs12817689	519/158/15	319/113/10	0.136/0.150	0.78 (0.54–1.12)	0.173	1.10 (0.38–3.21)	0.865	0.85 (0.62–1.16)	0.311
	rs11067233	562/122/7	329/118/8	0.098/0.147	0.50 (0.34–0.73)	<0.001	0.73 (0.20–2.67)	0.631	0.57 (0.40–0.80)	0.001

*MAF* minor allele frequency, *OR* odds ratio, *CI* confidence interval

^a^Major homozygote/heterozygote/rare homozygote

^b^Logistic regression with adjustment for sex, age and smoking

Combined analyses were conducted to evaluate the cumulative effect of the three significant loci (Table 5). Intriguingly, we found a significant allele-dosage association between the number of favorable alleles and CHD risk (*P*_trend_ < 0.001). Compared with individuals carrying no favorable allele, those who carried “1–2” and “3–4” favorable alleles had lower risk of CHD with adjusted ORs of 0.71 (0.50–0.99) and 0.33 (0.18–0.58), respectively. Individuals carrying “3–4” favorable alleles had a 62 % (OR = 0.38, 95 % CI = 0.21–0.66) decreased risk of CHD compared with those carrying “0–2” favorable alleles.

**Table 5:** Combined analysis of the cumulative effect of rs11066782, rs11613718 and rs11067233 on coronary heart disease risk

Combined genotypes^a^	Cases	Controls	OR (95 % CI)^b^	*P* ^b^
0	383	222	1.00	
1–2	244	166	0.71 (0.50–0.99)	0.045
3–4	33	55	0.33 (0.18–0.58)	<0.001
*P* for trend				<0.001
0–2	627	388	1.00	
3–4	33	55	0.38 (0.21–0.66)	<0.001

*OR* odds ratio, *CI* confidence interval

^a^The combined genotypes were according to favorable alleles carried (rs11066782-T, rs11613718-T and rs11067233-G were considered as favorable alleles)

^b^Logistic regression with adjustment for sex, age and smoking

We also performed stratification analyses for the effect of rs11066782, rs11613718 and rs11067233 based on age, sex and smoking status (Additional file 3: Table S1). In ≥48 years group, rs11066782, rs11613718 and rs11067233 were significantly associated with a decreased risk of CHD after adjustment for the other covariates (OR = 0.70, 95 % CI = 0.50–0.97; OR = 0.71, 95 % CI = 0.51–0.99 and OR = 0.55, 95 % CI = 0.39–0.79, respectively). Sex stratification analysis indicated that rs11613718 had statistically associated with CHD in male (OR = 0.61, 95 % CI = 0.38–0.98) while rs11067233 had statistically associated with CHD in female (OR = 0.35, 95 % CI = 0.20–0.62) after adjustment for covariates respectively. Besides, rs11067233 was significantly associated with a decreased risk of CHD in never-smokers after adjustment for relevant covariates (OR = 0.37, 95 % CI = 0.23–0.59).

## Discussion

In this study, we investigated the relationship of 17 SNPs located in three genes (*MVK*, *MMAB* and *KCTD10*) with HDL-C concentrations and CHD risk in a Chinese population. We found that rs11067233 in *MMAB*, rs11066782 and rs11613718 in *KCTD10* were associated with HDL-C concentrations and with CHD risk.

The *KCTD10* gene, located on chromosome 12q24, is a member of the polymerase delta-interacting protein 1 gene family [20]. Reports showed that *KCTD10* was highly expressed in human heart, skeletal muscle, and placenta, and play a vital role in DNA synthesis by interacting with proliferating cell nuclear antigen and polymerase δ [21]. In A549 lung adenocarcinoma cells, down-regulation of *KCTD10* could inhibit cell proliferation [22]. Transcription factors SP1 and AP-2α could bind to the promoter region of human *KCTD10*, and regulate its expression [23]. A recent study revealed *KCTD10* as a novel prognostic biomarker in gastrointestinal stromal tumor [24]. According to Chen et al., Murine *Kctd10* has been implicated in a metabolic network perturbed by loci contributing to the susceptibility of obesity, diabetes, and atherosclerosis [18]. And by extension, the involvement of human *KCTD10* in this metabolic network supports its association with HDL-C concentrations and CHD development. In the present study, we found, for the first time, that the minor alleles of rs11066782 and rs11613718 in *KCTD10* were associated with higher HDL-C concentrations and lower CHD risk in a Chinese population. Since high levels of HDL-C could reduce the risk of ischaemic heart disease [8, 25], it is reasonable that the variant genotypes of the two polymorphisms may affect CHD susceptibility by increasing HDL-C concentrations. Located at intron 1 of *KCTD10* gene, the two SNPs are in high linkage disequilibrium with each other (*r* ^2^ = 0.92), and both of them may affect gene expression by altering transcription factor binding sites (TFBS) of *KCTD10* based on SNPinfo Web Server (http://snpinfo.niehs.nih.gov/). Analysis of Encyclopedia of DNA Elements data as implemented in online tool, RegulomeDB (http://regulomedb.org/), indicated that the two polymorphisms may influence the histone modifications and the expression of *KCTD10*, which may serve as regulatory variants in the development of coronary heart disease. Moreover, data from GTEx (http://www.gtexportal.org/home/) revealed that variant genotypes of the two potential SNPs were significantly associated with higher expression levels of *KCTD10* in liver tissues in all HapMap subjects (*P* = 0.041 for rs11066782 and *P* = 0.038 for rs11613718, respectively; Additional file 4: Figure S2a, b). Besides, stratified analyses subsequently demonstrated that the protective effects of the variant genotypes were greater in those aged ≥48 years for rs11066782 and rs11613718, and greater in men for rs11613718 compared with other individuals. Since the prevalence of CHD increases with advancing age [26, 27] and is greater in men than women [28], it is reasonable that such genetic effects are more evident among the older people and men. Nevertheless, larger sample size and multi-ethnic population studies are warranted to support our findings.

In humans, *MVK* and *MMAB* are arranged in a head-to-head orientation on chromosome 12. Through a shared common promoter, *MVK* and *MMAB* are both regulated by sterol-responsive element-binding protein 2 (SREBP2), which is a transcription factor that controls cholesterol homeostasis [17]. Furthermore, it is also reported that these two neighboring genes participate in metabolic pathways associated with HDL metabolism. *MVK* encodes for mevalonate kinase, which catalyzes an early step in the biosynthesis of cholesterol [17]. Homozygosity for milder mutations in *MVK* could cause hyperimmunoglobulinemia D syndrome (HIDS), which is characterized by fever and increased levels of immunoglobulin D and A [29, 30]. In keeping with GWAS findings [13, 14], patients with HIDS have low HDL-C concentrations. However, in our study, neither rs3759387 in *MVK* nor rs2287218 in *MVK* showed a consistent result with the previous studies. The effects of SNPs on dyslipidemia might differ according to different ethnic background, as the rs3759387 T allele and rs2287218 T allele were found to be more enriched in the Caucasians than in Asians; Additionally, these discrepancies may be due to differences in lifestyle across populations. Functional characterizations are warranted to determine the causal variants related to plasma lipid concentrations in Asian populations. *MMAB* encodes the cob(I)alamin adenosyltransferase, an enzyme involved in the formation of adenosylcobalamin, necessary for degradation of cholesterol [17]. In humans, deficiency of cob(I)alamin adenosyltransferase results in methylmalonic aciduria [31], and a negative correlation between urinary methylmalonic acid and red blood cell membrane cholesterol concentrations was also found in patients with schizophrenia [32]. According to Junyent et al., homozygotes for major alleles at SNPs *MMAB* 3U3527G > C displayed lower HDL-C concentrations than carriers of the minor alleles when they consumed diets rich in carbohydrates [33]. Besides, Marie et al. observed significant allelic expression imbalance of 22 % in *MMAB* (transcribed SNP rs11067231) and the allele associated with lower HDL-C level displayed greater *MMAB* transcript level. This result indicated that *MMAB* was a likely susceptibility gene influencing HDL-C levels [34]. In addition, *MMAB* can affect TG levels through adenosylcobalamin, methylmalonyl-CoA mutase [35] and homocysteine [36, 37] based on several related function researches. In this study, we found that the variant genotypes of rs11067233 were associated with higher HDL-C concentrations and lower CHD risk. According to SNPinfo Web Server, rs11067233 may affect *MMAB* gene expression by altering microRNA binding at the 3′UTR of *MMAB*. Meanwhile, rs11067233 C > G could result in the loss of hsa-miR-603 binding to *MMAB* with energy change of 20.90, as predicted by miRNASNP v2.0 (http://bioinfo.life.hust.edu.cn/miRNASNP2/), which is a online database that predicts the effect (loss or gain of function) of miRNA-related SNPs based on miRNA expression data, GWAS information and experimental validation results. Additionally, data from GTEx project suggested that the variant genotypes of rs11067233 showed relatively lower expression levels of *MMAB* in liver tissues compared with homozygotes, although the difference did not reach the statistical significance level (*P* = 0.078; Additional file 4: Figure S2c). Moreover, Fogarty et al. also found that the C allele of rs11067233 demonstrated 15 % higher *MMAB* expression than the G allele in human hepatocytes [34]. Taken together, it is plausible that rs11067233 is associated with *MMAB* gene expression, which therefore could alter serum lipid concentrations, and accordingly modify the risk of coronary artery disease. Notably, we found that rs11067233 genotypes were significantly associated with lower risk of CHD among never-smokers and females. As cigarette use is a well-established risk factor for CHD [38, 39] and most of the never-smokers in China are females, the protective effect of rs11067233 may be diluted by environmental risk factors if they do not have a joint effect. Anyhow, further investigations in relation to biological mechanisms may lead to important insights for these findings.

Several limitations of our study should be considered. Though we observed significantly main effects of three polymorphisms on CHD risk, only rs11067233 remained significant after Bonferroni correction for multiple comparisons (*P* < 0.010, Bonferroni-adjusted), suggesting that larger well-designed studies are warranted to confirm the associations identified in our study. Secondly, the sample size was moderate with limited power to detect significant associations. Thirdly, inherent selection bias could not be completely excluded due to the unbalanced matching in age and sex. Finally, some other confounding factors may potentially mediate the effect of selected polymorphisms on dyslipidemia and CHD risk such as alcohol consumption, physical activity, family history of cardiovascular diseases and so on. However, we applied a rigorous epidemiological design and laboratory tests and statistically adjusted for several known risk factors to minimize potential bias.

## Conclusions

In conclusion, we found that rs11067233 in *MMAB*, rs11066782 and rs11613718 in *KCTD10* were associated with higher HDL-C concentrations and lower CHD risk in a Chinese population. Further studies incorporating diverse populations and functional assays are required to validate and extend these findings.

## Additional files

Additional file 1: Figure S1. The LD figure for the 18 loci included in this study. (DOCX 112 kb)
Additional file 2: The dataset of this article. (CSV 145 kb)
Additional file 3: Table S1. Stratification analysis on the associations of rs11066782, rs11613718 and rs11067233 with coronary heart disease risk. (DOCX 16 kb)
Additional file 4: Figure S2. Box plots showing gene expression levels by different variant genotypes identified from GTEx database. (DOCX 101 kb)

## Abbreviations

BMI: Body mass index
CHD: Coronary heart disease
CI: Confidence interval
GWAS: Genome-wide association study
HDL-C: High-density lipoprotein-cholesterol
LD: Linkage disequilibrium
LDL-C: Low-density lipoprotein-cholesterol
MAF: Minor allele frequency
OR: Odds ratio
PCR: Polymerase chain reaction
SNP: Single nucleotide polymorphism
TC: Total cholesterol
TFBS: Transcription factor binding sites
TG: Triglycerides
