# Supplementary figures and images for: Association of KCTD10, MVK, and MMAB polymorphisms with dyslipidemia and coronary heart disease in Han Chinese population
Source: Lipids Health Dis. 2016 Oct 4;15:171. doi: 10.1186/s12944-016-0348-7 (PMC5050677; doi:10.1186/s12944-016-0348-7)

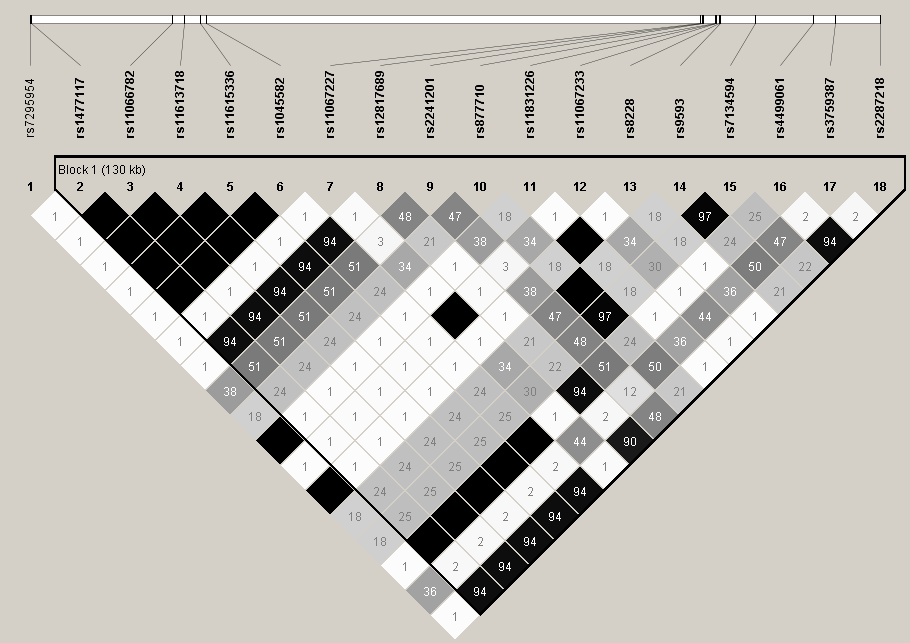


**Figure S1** The LD figure for the 18 loci included in this study

Supplement: Additional file 1: Figure S1. — The LD figure for the 18 loci included in this study. (DOCX 112 kb) [file 12944_2016_348_MOESM1_ESM.docx]
