# Supplementary material for: Association of KCTD10, MVK, and MMAB polymorphisms with dyslipidemia and coronary heart disease in Han Chinese population
Source: Lipids Health Dis. 2016 Oct 4;15:171. doi: 10.1186/s12944-016-0348-7 (PMC5050677; doi:10.1186/s12944-016-0348-7)
Supplement: Additional file 3: Table S1. — Stratification analysis on the associations of rs11066782, rs11613718 and rs11067233 with coronary heart disease risk. (DOCX 16 kb) [file 12944_2016_348_MOESM3_ESM.docx]

**Table S1** Stratification analysis on the associations of rs11066782, rs11613718 and rs11067233 with coronary heart disease risk

| Variables | rs11066782 | | rs11613718 | | rs11067233 | |
| --- | --- | --- | --- | --- | --- | --- |
|  | OR (95% CI) ^a^ | *P* ^b^ | OR (95% CI) ^a^ | *P* ^b^ | OR (95% CI) ^a^ | *P* ^b^ |
| Age |  | 0.655 |  | 0.681 |  | 0.990 |
| <48 | 0.86(0.37-2.00) |  | 0.86(0.37-2.01) |  | 0.55(0.20-1.53) |  |
| ≥48 | 0.70(0.50-0.97) |  | 0.71(0.51-0.99) |  | 0.55(0.39-0.79) |  |
| Sex |  | 0.843 |  | 0.602 |  | 0.078 |
| Male | 0.63(0.39-1.01) |  | 0.61(0.38-0.98) |  | 0.70(0.42-1.17) |  |
| Female | 0.67(0.41-1.10) |  | 0.73(0.45-1.19) |  | 0.35(0.20-0.62) |  |
| Smoking |  | 0.727 |  | 0.484 |  | 0.028 |
| Never | 0.68(0.45-1.02) |  | 0.73(0.48-1.10) |  | 0.37(0.23-0.59) |  |
| Ever | 0.59(0.32-1.10) |  | 0.56(0.30-1.04) |  | 0.89(0.47-1.67) |  |

*OR* odds ratio, *CI* confidence interval

^a^ Logistic regression with adjustment for sex, age and smoking except for the stratification factor

^b^ Heterogeneity test for differences between groups
