# Supplementary material for: Association of KCTD10, MVK, and MMAB polymorphisms with dyslipidemia and coronary heart disease in Han Chinese population
Source: Lipids Health Dis. 2016 Oct 4;15:171. doi: 10.1186/s12944-016-0348-7 (PMC5050677; doi:10.1186/s12944-016-0348-7)
Supplement: Additional file 4: Figure S2. — Box plots showing gene expression levels by different variant genotypes identified from GTEx database. (DOCX 101 kb) [file 12944_2016_348_MOESM4_ESM.docx]

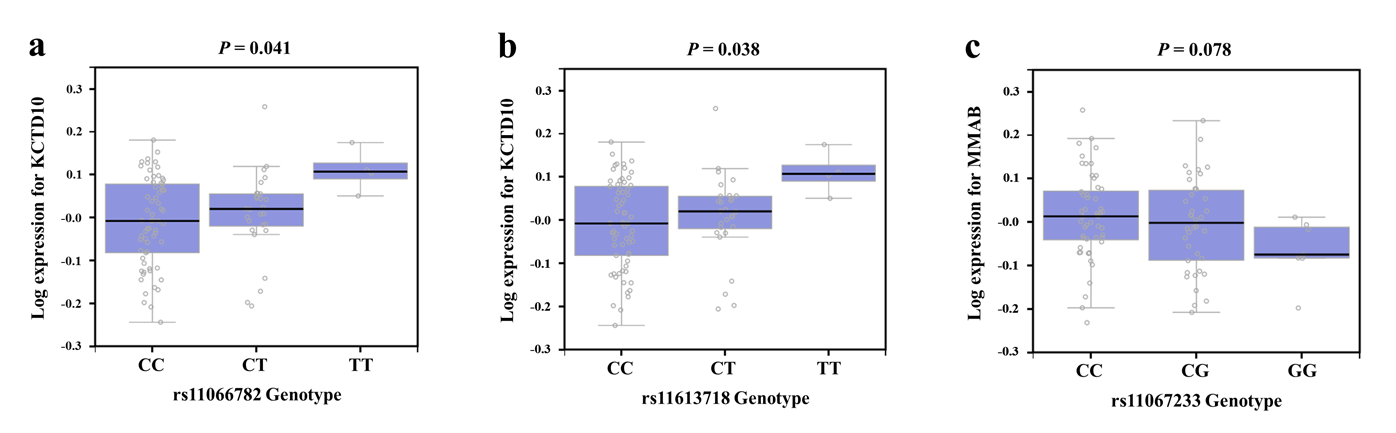


**Figure S2** Box plots showing gene expression levels by different variant genotypes identified from GTEx database. The three genotype class categories of every polymorphism are shown on the horizontal axis, and the vertical axis indicates the log normalized expression value of certain gene. The vertical bars below and above the box indicate the minimum and maximum values, respectively; the lower and upper limits of the box are the 25th and 75th percentiles, respectively, and the line inside each box is the median. a *KCTD10* expression levels by rs11066782 genotypes; b *KCTD10* expression levels by rs11613718 genotypes; c *MMAB* expression levels by rs11067233 genotypes
